# Recent Advances in Combinatorial Optimization

**DOI:** 10.1155/2015/628265

**Published:** 2015-04-01

**Authors:** Dehua Xu, Dar-Li Yang, Ming Liu, Feng Chu, Imed Kacem

**Affiliations:** ^1^College of Science, East China Institute of Technology, Nanchang, Jiangxi 330013, China; ^2^Department of Information Management, National Formosa University, Yun-Lin 632, Taiwan; ^3^School of Economics & Management, Tongji University, Shanghai 200092, China; ^4^Laboratoire IBISC, Université d'Evry-Val d'Essone, 40 rue Pelvoux, 91001 Evry, France; ^5^LCOMS EA7306, Université de Lorraine, Ile du Saulcy, 57000 Metz, France

Combinatorial optimization is one of the most active branches of operations research. The essence of a combinatorial optimization problem is to find optimal solutions or near optimal solutions from a finite set of feasible solutions. In such problems, the size of feasible solution space usually increases exponentially with regard to the increase in the size of the input parameters. This issue, which has an acceptance rate of less than 30%, compiles six exciting papers.

In the paper “Single Machine Scheduling and Due Date Assignment with Past-Sequence-Dependent Setup Time and Position-Dependent Processing Time,” by C.-L. Zhao et al., the authors study several objective functions including total earliness, the weighted number of tardy jobs, and the cost of due date assignment. They provide polynomial time algorithms for all the considered problems. In the paper “Scheduling Jobs and a Variable Maintenance on a Single Machine with Common Due-Date Assignment,” by L. Wan, the author derives some properties on an optimal solution for the problem and proposes an optimal polynomial time algorithm for a special case with identical jobs. In the paper “Due-Window Assignment Scheduling with Variable Job Processing Times,” by Y.-B. Wu and P. Ji, the authors prove that the problem can be solved in polynomial time. In the paper “Some Single-Machine Scheduling Problems with Learning Effects and Two Competing Agents,” by H. Li et al., the authors investigate three problems arising from different combinations of the objectives of the two agents. They provide a polynomial time algorithm for one problem and two polynomial time algorithms for the other two problems under certain agreeable conditions. In the paper “An Order Insertion Scheduling Model of Logistics Service Supply Chain Considering Capacity and Time Factors,” by W. Liu et al., the authors analyze order similarity coefficient and order insertion operation process and establish an order insertion scheduling model of LSSC with service capacity and time factors considerations. In the paper “Cooperative Fuzzy Games Approach to Setting Target Levels of ECs in Quality Function Deployment,” by Z. Yang et al., the authors develop a cooperative game framework combined with fuzzy set theory to determine the target levels of the engineering characteristics in quality function deployment.

The papers published in this issue contain some interesting, creative, and valuable results and ideas. We do believe that all these papers will motivate further scientific research in combinatorial optimization and related areas.

*Dehua Xu* *Dehua Xu* *Dar-Li Yang* *Dar-Li Yang* *Ming Liu* *Ming Liu* *Feng Chu* *Feng Chu* *Imed Kacem* *Imed Kacem*

